# Leucinostatins target *Plasmodium* mitochondria to block malaria transmission

**DOI:** 10.1186/s13071-024-06608-8

**Published:** 2024-12-20

**Authors:** Guodong Niu, Xiaohong Wang, Jun Li

**Affiliations:** Department of Biological Sciences, Florida International University, 11200 SW 8th St, Miami, FL 33199 USA

**Keywords:** Malaria, Leucinostatin, Transmission-blocking, Mitochondria, MOA, Molecular mechanism, Mechanism of action

## Abstract

**Background:**

Malaria remains a critical disease. Leucinostatins from the fungus *Purpureocillium lilacinum* inhibited the transmission of *Plasmodium falciparum* to mosquitoes via contact.

**Methods:**

Here, we modified the leucinostatin B (LB) C-terminus to make derivatives and examined their inhibition against malaria transmission to mosquitoes. Fluorescence-labeled leucinostatins were incubated with intact gametocytes and were examined under microscopy to detect the targets of leucinostatins. We also analyzed leucinostatins’ general cytotoxicity and hemolysis.

**Results:**

The results showed that the derivatives with –H, –CH_3_, –Atto495, and –Biotin at C-terminus had EC_50_ of 1.5 nM, 0.2 nM, 4.2 nM, and 42 nM, respectively. Atto495 and biotin are similar in size and much bigger than -CH_3_ and -H. Based on reverse-phase HPLC elution time, we found that LB-Biotin had much higher hydrophobicity than the others, consistent with its lowest malaria transmission-blocking activity. Fluorescence microscopy showed that LB-Atto495 colocalized with mitochondria inside intact *P*. *falciparum* gametocytes. We found that leucinostatin A significantly inhibited the proliferation of human nucleated cells with IC_50_ around 47 nM and it did not lyse erythrocytes at 100 μM.

**Conclusions:**

We conclude that the leucinostatins pass through the cytoplasmic membrane without lysing cells and interact with molecules specifically in mitochondria. Therefore, leucinostatins should be ideal inhibitors against mobile parasites, such as ookinetes and sporozoites, during malaria transmission.

**Graphical Abstract:**

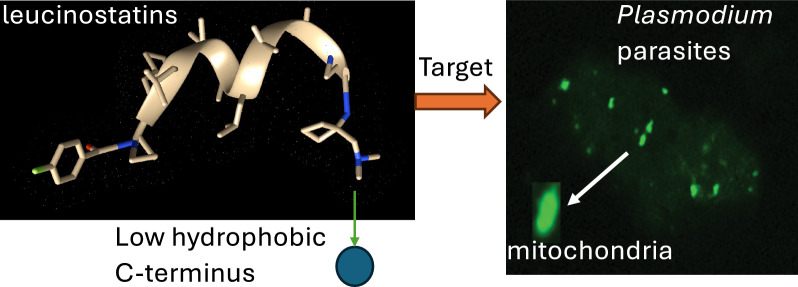

## Background

According to the world malaria report, there were still 241 million malaria cases and 627 thousand malaria deaths worldwide in 2023. The resistance of *Plasmodium* parasites to artemisinin [1], widespread resistance of mosquitoes to pyrethroids [2], and the modest efficacy of malaria vaccine RTS, S (Mosquirix) [3] make malaria control a challenging task. Therefore, malaria communities urgently need novel strategies for malaria control.

Compared with traditional methods against malaria, transmission-blocking has been considered for its great potential in malaria control. From a diverse global fungal extract library [4], several drug leads with great TB capacities have been identified [5–7]. Most recently, the ethyl acetate extract of the fungus *Purpureocillium lilacinum* was discovered to inhibit *P*. *falciparum* transmission to mosquitoes [7]. Notably, pre-exposure of the *P*. *lilacinum* extract to mosquitoes significantly reduces *P*. *falciparum* oocysts in mosquito midguts [7], suggesting that the fungal extracts can be sprayed like insecticides to prevent malaria transmission. Furthermore, we isolated the active small molecules, leucinostatins, from *P*. *lilacinum*, which blocked malaria transmission to mosquitoes and reduced sporozoites in mosquito salivary glands [8].

Leucinostatins have antimicrobial activity against gram-positive bacteria, including *Staphylococcus albus*, *Bacillus subtilis*, *Sarcina letter*, *Streptococcus pyrogens*, *Streptococcus faecalis*, and Mycobacterium species, with IC_50_ values ranging from 2.5 to 100 μM and against some fungal strains with minimal inhibitory concentrations of 10–25 μM [9–11]. In addition, leucinostatins are known as mycotoxins. The IC_50_ of leucinostatin Α (LA) to MRC-5 (human fetal lung fibroblast cells) is 2 μΜ [12]. Leucinostatins also inhibited the proliferation of varying cancer cells. The IC_50_ of LA against Hela cells is about 40 nM [9], and the IC_50_ of LA against six triple-negative breast cancer cell lines is between 10 and 100 nM [13]. Compared with normal cells, cancer cells are more sensitive to leucinostatins.

The in vivo toxicity of leucinostatins on mice indicated that the LD_50_ of LA via intraperitoneal injection was 1.6 mg/kg [9]. In comparison, Mikami et al. reported an LD_50_ via i.p. of 1.8 mg/kg and 5.4 to 6.3 mg/kg by oral administration in a single dose [10].

Leucinostatins are non-ribosomal peptides with non-standard amino acids. Unlike regular peptides, leucinostatins are synthesized through individual enzymes encoded within a gene cluster [14]. LB contains nine amino acid residues, including the unusual amino acid 4-methyl-L-proline (MePro), 2-amino-6-hydroxy-4-methyl-8-oxodecanoic acid (AHyMeOA), hydroxyleucine (HyLeu), α-aminoisobutyric acid (AIB), β-Ala, and a 4-methylhex-2-enoic acid at the N-terminus as well as an N1-methylpropane-1,2-diamine (DPD) at the C-terminus. To date, 24 different structures have been described in the leucinostatin series, which differ in the amino acid at position at position 2 (Dec or Leu) and in the substitution pattern at the terminal nitrogen atom [–N(CH3)2, –NHCH3, –NH2, or –NO(CH3)2]. The unique composition of the peptides makes leucinostatins very hydrophobic and tend to form helixes.

Since this novel strategy for malaria control could play an important role in malaria elimination and eradication programs, we studied the mode of action here. First, we investigated their structures and activities in this study. Previous reports show that changing the LB N-terminus altered its activities and specificity [15]. Here, we modified the LB C-terminus with different sizes and hydrophobicity and examined their malaria transmission-blocking activity. We also determined the leucinostatins’ targets in cells and their general cytotoxicity and cytolyses to understand the mechanisms of action against malaria.

## Methods

### Mosquito maintenance

As we described previously [16], we maintained *An*. *gambiae* (G3 strain) at 27 °C with 80% humidity and a 12 h day/night cycle. Larvae were fed fish food for koi, adult mosquitoes were maintained with 10% sugar in water, and female mosquitoes were fed with purchased human blood to lay eggs. Eggs were collected 48 h after blood-feeding with wet filter papers and hatched in pure water.

### *P*. *falciparum* culture and standard membrane feeding assays (SMFA)

*P*. *falciparum* (NF54) was cultured in the lab [7, 8, 17] with 4% O + -type red blood cells in a complete Roswell Park Memorial Institute (RPMI) 1640 medium with 10% human AB + serum and 12.5 μg/ml of hypoxanthine in a candle jar at 37 °C. Then, 15-day-old cultured *P*. *falciparum*-infected cells were collected through centrifugation (300 *g* for 3 min), and about 100 μl of pellet were resuspended in 1.5 ml fresh AB + -type serum and mixed with 1.4 ml O + -type red blood cells, containing about 0.2% stage V gametocytes [16]. Each candidate compound was dissolved in dimethylsulfoxide (DMSO) and diluted with DMSO to obtain different concentrations. About 2 μl of candidate solution was mixed with 298 μl of *P*. *falciparum*-infected blood prepared above and was used to feed 60–80 3–5-day-old *An*. *gambiae* with feeders covered with parafilm at 37 °C for 30 min. The fed mosquitoes were maintained in an insectary with 10% sugar in water for 7 days in the insectary and dissected to obtain midguts. The midguts were stained with 0.1% mercury dibromofluorescein disodium salt in phosphate-buffered saline (PBS), and the oocysts were counted under a light microscope.

### Modifying LB C-terminus

LA and LB were purified from *P*. *lilacum* as described [18]. LA/LB mixture (Sigma) was also purchased from Sigma. LB C-terminus were modified by linking biotin and Atto495. Around 0.4 µmol of LB and 8 µmol of amine-reactive EZ-Link™ NHS-LC-Biotin (Invitrogen; molar ratio: 1:20) were mixed in 2 mL of PBS buffer (pH = 7.2) and reacted for continuous stirring 4 h at room temperature. Then, the reaction sample was analyzed and purified by semi-prep high-performance liquid chromatography (HPLC; Shimadzu) C18 column (Gemini 5 μm 110A, 250 × 10 mm) with gradient elution of 50% methanol to 100% methanol in 50 min. The flow rate was 1 ml per min. The LB-biotin conjugate fraction was dried at room temperature with a rotary vacuum evaporator (Heidolph).

The reaction of LB with amine-reactive Atto495 NHS ester (Sigma) was conducted in DMSO. Firstly, Atto495 NHS ester (3 mM), LB (1 mM), and triethylamine (100 mM) were mixed in DMSO in a 0.2 mL reaction volume and reacted at room temperature for 4 h with continuous stirring while protected from light. The reaction solution was applied to semi-prep HPLC to obtain the conjugate of LB-Atto495 as described above, and the LB-Atto495 was dried for use.

### Detecting the distribution of LB in cells

The 15-day-old cultured *P*. *falciparum* was collected from a six-well plate. After centrifugation (300 *g* for 3 min), the cells were washed with 1 ml of PBS three times at room temperature. About 10 μl of cells was suspended in 100 μl of PBS, and 1 μl of LB-Atto495 (1 mM in DMSO) was added and mixed. The cells were incubated at room temperature in the dark for 6 h. The mixture was centrifuged at 300 *g* for 3 min to collect cells, which were washed three times with 200 μl of PBS. Finally, the cells were deposited on slides to make a blood smear and fixed with methanol. The vector shield antifade mounting medium for fluorescence (VectorLabs, CA) was dropped on the slides. The slides were covered with coverslips and examined under a fluorescent microscope (Nikon Eclipse Ti-S).

### Cytotoxicity assays

Two methods and two types of cells were used to analyze the general cytotoxicity of small molecules. First, MTT (3-(4,5-dimethylthiazol-2-yl)-2,5-diphenyl tetrazolium bromide; Thermo-fisher) and human embryonic kidney 293 (HEK293) were used in cytotoxicity assays. About 20,000 HEK293 cells in 200 µl of culture medium [Dulbecco’s modified eagle medium (DMEM) with high glutamine and 10% fetal bovine serum] were seeded per well in 96-well microplates. About 1 µl of the compounds in DMSO at various dilutions was added into each well to obtain final concentrations of 0, 10, 100, and 1000 ng/mL. Three replicates were conducted for each concentration. Following incubation at 37 °C with 5% CO_2_ for 24 h, 10 µL of MTT (5 mg/mL in PBS) was added to each well and incubated for 4 h at 37 °C with 5% CO_2_. All but 25 µL of the medium was removed from the wells, and 100 µL of DMSO was added to each well and incubated at 37 °C for 10 min to dissolve formazan crystals for measurement. Optical density was measured at an absorbance wavelength of 540 nm.

Second, the CCK-8 kit (Sigma) and K562 cell line (human immortalized myelogenous leukemia cell) were used to determine the inhibition of a compound against cell proliferation. About 20,000 cells in 200 µl of culture medium (RMPI-1640 with 10% fetal bovine serum) were seeded per well in 96-well microplates, and then a series of dilutions of the compound from 0 to 1000 ng/ml was added to the medium. The cells were maintained at 37 °C and 5% CO_2_ for 48 h, and then 20 μl of the CCK-8 solution was added to each well of the plate. The plate was incubated for 1 h, and the A_450_ was measured using a microplate.

### Hemolysis assays

About 200 µL of O + human red blood cells were washed three times in PBS (pH 7.4) and then suspended with 1 ml of PBS. Then, 0.3 ml of suspension was diluted with 3 mL of PBS to obtain a 10% suspension. All tested compounds were dissolved in DMSO to prepare a series of dilutions of 1 nM, 10 nM, 100 nM, 1 µM, 10 µM, and 100 µM. The erythrocyte suspension (100 μl per well) was added to a 96-well plate, and the samples were rapidly stirred and incubated at 37 °C with periodic stirring during a 3-h incubation period. The solution was then centrifuged at 2,000 rpm for 5 min. The absorbance of the supernatant was measured at 540 nm using a microplate, and the hemolytic ratio (%) was calculated by comparison with the 100% hemolytic activity caused by distilled water as maximal hemolytic control. The hemolytic % developed by the PBS control was subtracted from all groups. Each experiment was performed in triplicate.

### Statistical analysis

The experiment was performed three times independently. Data were analyzed using Prism 8 (GraphPad Software, CA). Since the number of oocysts in mosquito midguts does not follow a normal distribution, the Wilcoxon-Mann–Whitney test was used to obtain the *p*-value. For the rest of the experiments, the *t*-test and analysis of variance (ANOVA) were used to calculate the *p*-value.

## Results

### Modification of leucinostatins

**Fig. 1:**
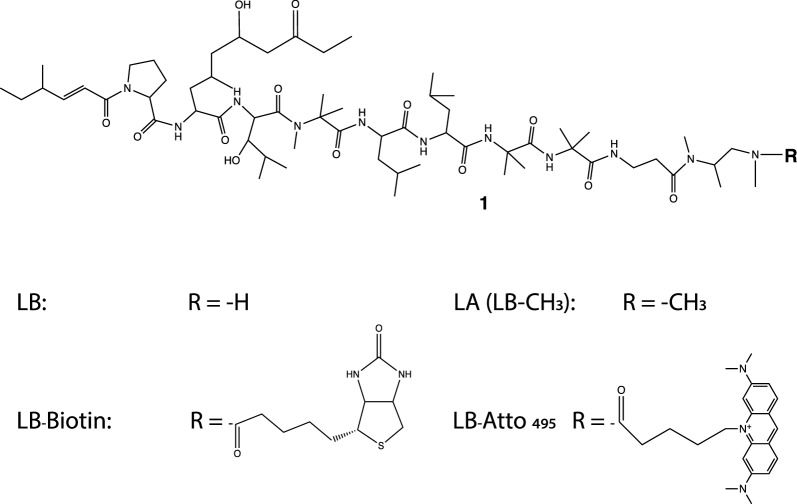
The structure of modified LB

The size and hydrophobicity of the LB C-terminus were modified with -CH_3_ (LA), -biotin, and Atto495. Their structures are shown in Fig. 1. LB has an amine at its C-terminus, where chemical groups are linked by a covalent bond. The sizes of four chemicals, in ascending order, are LB < LA < LB-Atto495 ≈ LB-biotin.

**Fig. 2:**
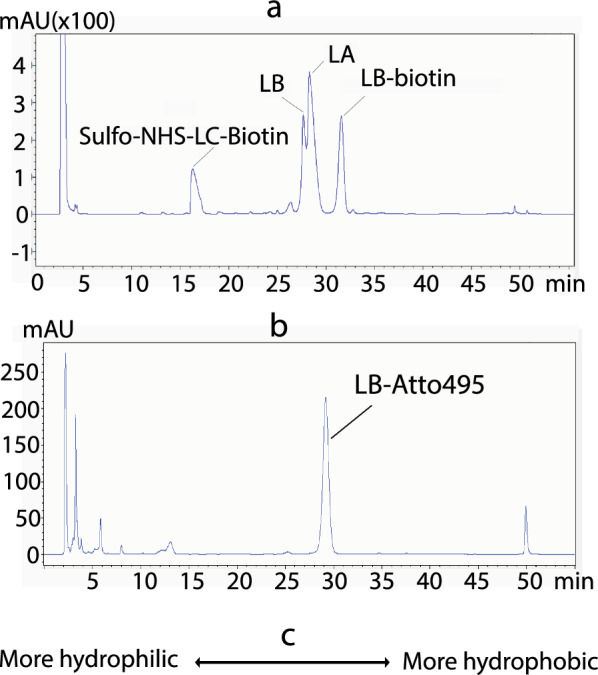
Generation and purification of LB derivatives. **a** HPLC elution profile of LA, LB, LB-biotin, and sulfo-NHS-LC-Biotin after the reaction. **b** HPLC elution profile of LB-Att495. **c** The relationship between the elution time and hydrophobicity

LA/LB was mixed with EZ-Link™ NHS-LC-Biotin. After the reaction, semi-prep HPLC was used to purify LB-biotin. From hydrophilic to hydrophobicity, sulfo-NHS-LC-Biotin, LB, LA (LB-CH_3_), and LB-biotin were eluded at the approximate time of 16.5, 27.5, 28.3, and 31.5 min, sequentially (Fig. 2a). Based on the A_254_, about half of the LB was transformed into LB-biotin.

The pure LB was reacted with amine-reactive Atto495 NHS ester in DMSO. The reaction solution was applied to semi-prep HPLC. The conjugate of LB-Atto495 was eluded at 29 min (Fig. 2b). When a reverse phase C18 column was used, compounds with higher hydrophobicity exhibited stronger interactions with the stationary phase and eluted later, while less hydrophobic compounds eluted earlier. On the basis of elution times from the HPLC C18 column, the hydrophobicity of four compounds is LB < LA < LB-Atto495 < LB-biotin.

### The malaria transmission-blocking activity of LB and its derivatives

**Fig. 3:**
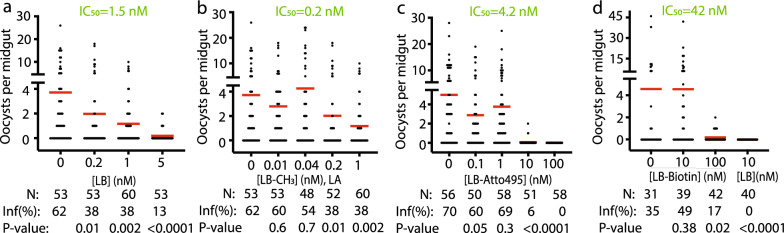
Malaria transmission-blocking activity of leucinostatins by SMFA. **a** The TB activity assays of LB. **b** The TB activity assay of LA. **c** The TB activity assay of LB-Atto495. **d** The TB activity assay of LB-biotin at different concentrations and LB at 10 nM

After modifying the LB C-terminus, we analyzed their activity in blocking malaria transmission to mosquitoes using SMFA. The results show that all four small molecules inhibited *P*. *falciparum* transmission to mosquitoes at sub-micromolar concentration (Fig. 3). The IC_50_ of LB, LA, LB-Atto495, and LB-biotin were calculated to be 1.5 nM, 0.16 nM, 4.2 nM, and 42 nM, respectively. The inhibition was concentration-dependent. Therefore, the order of activity from high to low is LB-biotin < LB-Atto495 < LB < LA. Modifying LB with Atto495 did not change LB TB activity (Fig. 3c). We also compared the activity of LB and LB-biotin at 10 nM using the same set of cultured parasites and mosquitoes. Results show that 10 nM LB completely blocked *P*. *falciparum* transmission to mosquitoes, while 10 nM LB-biotin did not inhibit malaria transmission (Fig. 3d).

### The target of leucinostatin in *P*. *falciparum*

**Fig. 4:**
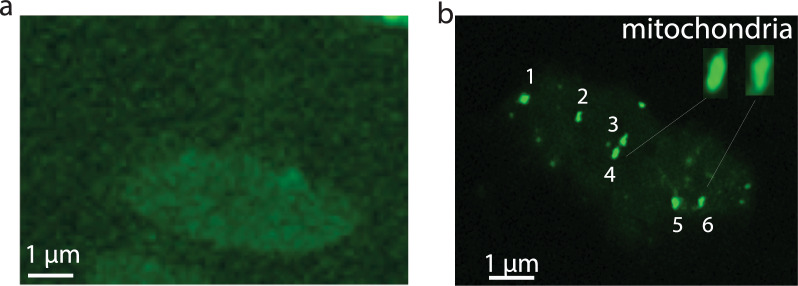
Leucinostatins colocalize with the *P*. *falciparum* mitochondria. Labeling LB with ATTO495 was incubated with live gametocytes and examined under a fluorescein microscope. **a** The control and **b** experiment

Next, we examined the binding location of leucinostatins. Since Atto495 is a fluorescent label, LB-conjugated Atto495 was incubated with live *P*. *falciparum* gametocytes. The cells on slides were observed using a fluorescent microscope. The Atto495 treated cells served as the negative control (Fig. 4a), showing no bright fluorescent spots. Results show that LB-Atto495 recognized some organelles in a *P*. *falciparum* gametocyte (Fig. 4b). Six large particles per gametocyte (Fig. 4b), which matches the number of mitochondria per gametocyte [19]. In addition, the shape of the particles is short rod-shaped or spherical (the insert in Fig. 4b), matching mitochondria [20]. Therefore, LB-Atto495 penetrated the living gametocyte cytoplasmic cell membrane and binds mitochondria specifically in parasites. In addition to mitochondria, some small irregular spots were also observed.

### The cytotoxicity assays of leucinostatins

**Fig. 5:**
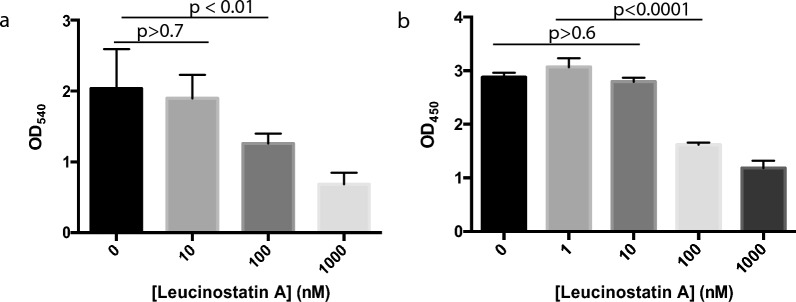
Cytotoxicity assay of leucinostatin A at different concentrations. **a** Using human embryonic kidney 293 (HEK293) cells and MTT assays. **b** Using human immortalized myelogenous leukemia (K562) cells and CCK-8 kit

Since leucinostatins target mitochondria, we analyzed their general cytotoxicity. LA (LB-CH_3_) is the most active compound; thus, we will use the cytotoxicity of LA as the representative. Two different cell lines, HEK293 and K562, were used to examine the general cytotoxicity of LA. The cells were incubated with LA to varying concentrations from 0 to 1000 nM. The results indicate no significant difference in the density of living cells between 0 and 10 ng/mL LA for HEK293 (*p* > 0.7; Fig. 5a) or K562 (*p* > 0.6, Fig. 5b). When the concentration was increased to 100 nM, LA reduced the cell proliferation by 38% for HEK293 and 43.7% for K562, and the corresponding IC_50_ was calculated as 89.6 nM and 47.3 nM, respectively. These IC_50_ values are the same level as that of LB-biotin in blocking malaria transmission but much higher than LA in blocking malaria transmission.

### Leucinostatins did not lyse red blood cells

**Fig. 6:**
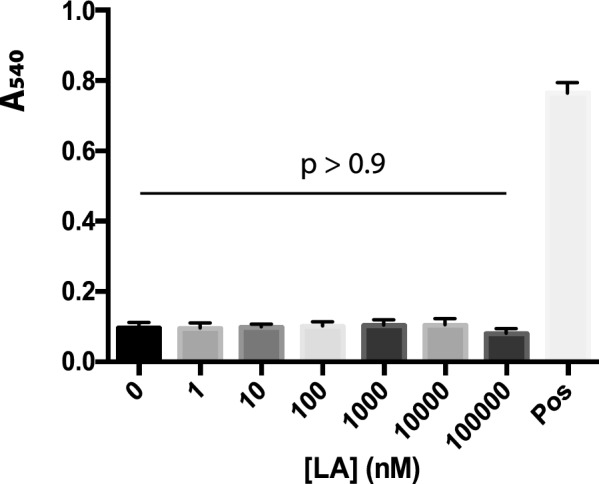
Hemolytic effects of leucinostatins. Pos: positive. RBCs were lysed in the water

The structure of LA was obtained from PDB (PBD ID: BA1A) [21]. LA forms α-helix and is hydrophobic. Therefore, we determined the hemolytic effect of LA. The red blood cells in water were used as the positive hemolytic control. Notably, at 100 µM concentration of LA, which was about 10^6^ times of IC_50_ of LA against malaria transmission, the hemolysis was not significantly different among the experimental group (*p* > 0.9) (Fig. 6). Therefore, it is unlikely that leucinostatins inhibit malaria transmission through disrupting cytoplasmic membrane.

## Discussion

Malaria remains a devastating disease, and infectious anopheline mosquitoes are essential for transmission. A recent report showed that mosquitoes contacting leucinostatins become resistant to malaria. Understanding their structure and function, toxicity, and mechanism is critical to applying leucinostatins in controlling malaria.

**Fig. 7:**
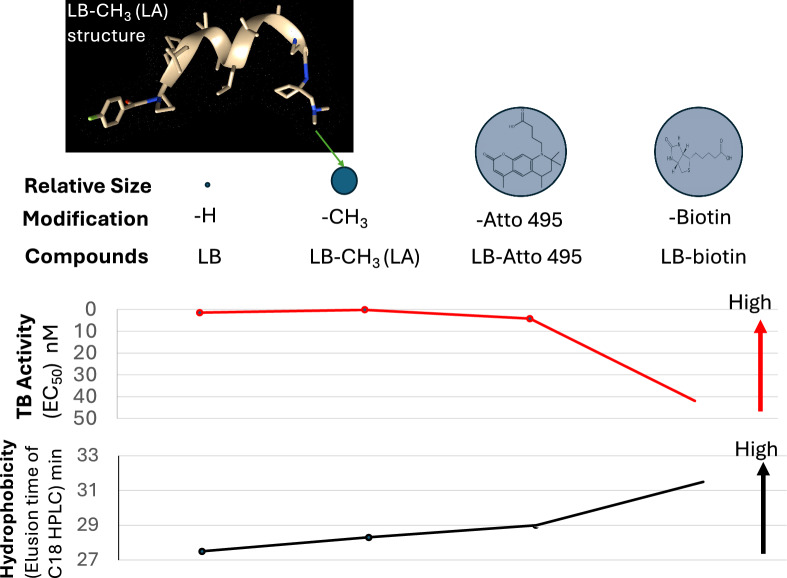
The modifications of LB C-terminus and their activities in blocking malaria transmission

We found that leucinostatins inhibited malaria transmission. Previous studies reported that leucinostatins are also toxic to mammalian cells [12, 13]. We analyzed leucinostatins to human cells, HEK293 and K562, and found the IC_50_ of LA to HEK293 and K562 are greater than 47 nM, more than 300 times the EC_50_ of LA to malaria transmission-blocking activity. Therefore, leucinostatins selectively target *P*. *falciparum*. Leucinostatins are hydrophobic with helix structures (Fig. 7); our data show that leucinostatins did not lyse the red blood, consistent with the results from mouse erythrocytes [22]. Our data support that leucinostatins penetrated the gametocyte cytoplasmic membrane without lysing cells and were enriched at the intracellular mitochondria.

Previous studies investigated the structure-activity relationships of leucinostatins. Brand et al. synthesized a series of leucinostatin derivatives and studied their functions. They found that the side chain of 2-amino-6-hydroxy-4-methyl-8-oxodecanoic acid (AHMOD) at the N-terminus of LA/LB is critical for their functions. The activity of LA/LB was improved five- to tenfold by modifying the N-terminus with an ethyl cyclohexyl or n-octyl sidechain. On the other hand, the activity is reduced when replacing the N-terminus with the hydrophilic glutamic acid or the hydrophilic n-butyl aliphatic chain [15]. Other studies found that replacing the residues of Aib, Leu, and AHMOD at positions 3, 8, and 10 reduced activity [23].

The C-terminus of leucinostatins has yet to be studied extensively. One report found that adding a carboxylic acid to the terminal amine on the C-terminus exhibits ten times less antimicrobial activity [24]. In this study, we modified the C-terminus of LB with four different chemical groups, varying with various sizes and hydrophobicity. LA, LB, and LB-Atto495, which differ in size but have similar hydrophobicity, exhibited strong inhibitory effects on parasites with EC_50_ around 1 nM (Fig. 7). LB-biotin is much more hydrophobic than the other three and was much less active than the other derivatives. Thus, the higher hydrophilic or hydrophobic C-terminuses substantially reduce leucinostatins’ TB activity against malaria.

Leucinostatins were reported to inhibit ATP synthesis in rat liver cell mitochondria [25]. The mixture of LA and LB has been observed to inhibit the coupling between the electron transport and phosphorylation reactions in rat liver mitochondria and submitochondrial particles, with inhibition of energy transfer at 240 nM [25]. Additionally, LA and its synthesized derivatives have been reported to interact with subunit c of mitochondrial ATP synthase by binding the essential carboxylate of Glu59, causing inhibition of activity in the DU-145 prostate cancer cell line [26]. However, leucinostatins did not inhibit the activity of the isolated F₁ part of the ATPase [27, 28]. Consistent with these reports, we observed that LB-Atto495 colocalized with mitochondria in *Plasmodium* gametocytes.

Moreover, LA caused distinct changes in mitochondrial ultrastructure in *Trypanosoma brucei* parasites [15]. Specifically, the mitochondrial matrix became less electron-dense, while other intracellular structures remained unaffected by the treatment [15]. These data support our conclusion that leucinostatins target *P*. *falciparum* mitochondria. Even though mitochondria are common organelles in nucleates, our data showed that the leucinostatins were 300 times more toxic to *Plasmodium* parasites than human cells. This selectivity suggests some differences between human cells and *Plasmodium*. Indeed, about 269 leucinostatin-derivative-binding proteins were pulled down from *T*. *gondii*, while 645 specific proteins were found in eluates from mouse spleen extracts [29].

We recently introduced a contact-wise inhibition of malaria transmission, demonstrating that leucinostatins could effectively limit *P*. *falciparum* infection in mosquitoes or transmission to humans if mosquitoes contact leucinostatins before or after blood-feeding [8]. As we reported previously, crude fungal extract from *Purpureocillium lilacinum* without purification could effectively inhibit the transmission of *Plasmodium falciparum* to mosquitoes via contact [7]. Since the fungus was isolated from soil [4], it is very cheap to culture it. Because mosquitoes become resistant to parasites after they contact the fungal extract, we could develop indoor antimalaria sprays. Given the poverty in malaria-endemic areas, this finding is significant because of the low cost and easy implementation. Although we do not intend to develop leucinostatins into antimalarial medicines, enhancing their activity or understanding their mode of action will guide their application.

In summary, leucinostatins are highly active against malaria transmission to mosquitoes. They play functions by targeting mitochondria specifically. The right hydrophobicity, e.g., -H and -CH_3_ at the C-terminus, is critical for leucinostatins’ inhibitory activity. Leucinostatins are ideal external reagents to block malaria transmission.

## Conclusions

We elucidated leucinostatins and derivatives for their contact-wise malaria transmission-blocking activity. We conclude that the leucinostatins pass through the cytoplasmic membrane without lysing cells and interact with molecules, specifically in mitochondria. During malaria transmission, leucinostatins should be ideal inhibitors against mobile parasites, such as ookinetes and sporozoites.

## Data Availability

No datasets were generated or analyzed during the current study.

## Abbreviations

LA: Leucinostatin A
SMFA: Standard membrane feeding assay
HPLC: High-performance liquid chromatography
TB: Transmission-blocking
